# Endurance training volume cannot entirely substitute for the lack of intensity

**DOI:** 10.1371/journal.pone.0307275

**Published:** 2024-07-22

**Authors:** Pekka Matomäki, Olli J. Heinonen, Ari Nummela, Heikki Kyröläinen

**Affiliations:** 1 Faculty of Sport and Health Sciences, University of Jyväskylä, Jyväskylä, Finland; 2 Paavo Nurmi Centre & Unit for Health and Physical Activity, University of Turku, Turku, Finland; 3 Finnish Institute of High Performance Sport KIHU, Jyväskylä, Finland; Universidad de León Facultad de la Ciencias de la Actividad Física y el Deporte: Universidad de Leon Facultad de la Ciencias de la Actividad Fisica y el Deporte, SPAIN

## Abstract

**Purpose:**

Very low intensity endurance training (LIT) does not seem to improve maximal oxygen uptake. The purpose of the present study was to investigate if very high volume of LIT could compensate the lack of intensity and is LIT affecting differently low and high intensity performances.

**Methods:**

Recreationally active untrained participants (n = 35; 21 females) cycled either LIT (mean training time 6.7 ± 0.7 h / week at 63% of maximal heart rate, n = 16) or high intensity training (HIT) (1.6 ± 0.2 h /week, n = 19) for 10 weeks. Two categories of variables were measured: Low (first lactate threshold, fat oxidation at low intensity exercise, post-exercise recovery) and high (aerobic capacity, second lactate threshold, sprinting power, maximal stroke volume) intensity performance.

**Results:**

Only LIT enhanced pooled low intensity performance (LIT: p = 0.01, ES = 0.49, HIT: p = 0.20, ES = 0.20) and HIT pooled high intensity performance (LIT: p = 0.34, ES = 0.05, HIT: p = 0.007, ES = 0.48).

**Conclusions:**

Overall, very low endurance training intensity cannot fully be compensated by high training volume in adaptations to high intensity performance, but it nevertheless improved low intensity performance. Therefore, the intensity threshold for improving low intensity performance is lower than that for improving high intensity performance. Consequently, evaluating the effectiveness of LIT on endurance performance cannot be solely determined by high intensity performance tests.

## Introduction

According to the overload principle, training below a minimum intensity fails to provide sufficient stimulus for the body to elicit increases in physical fitness [1]. However, a clear definition of the minimum intensity required for positive endurance performance adaptations remains elusive. For instance, the minimum training intensity to induce improvements in maximal aerobic capacity (VO_2max_) depends largely on the initial VO_2max_ level. Individuals with low initial capacity (<40 ml/kg/min) have no lower limit [2], while well-trained athletes are hypothesized to require training at a relatively high percentage, up to 95%, of their VO_2max_ to further enhance it [3]. A similar conclusion, suggesting that lower intensities are sufficient for improvement in individuals with lower initial values, has also been observed in lactate thresholds [4].

However, training intensity is not the sole factor governing adaptations in VO_2max_ as other factors including training frequency [5] and duration of exercise sessions [6] also impact the magnitude of these adaptations. Further, endurance performance is influenced by various variables, which are categorized in this study into low- and high intensity performance categories. *Low intensity performance category* encompasses all variables that impact endurance performance measured below the first lactate threshold (LT_1_), such as fat oxidation capacity [7], durability [8], economy of movement [9] and post exercise recovery [10]. *High intensity performance category* refers to endurance performance related variables that are measured above LT_1_, include variables such as VO_2max_, second lactate threshold (LT_2_) [9], maximal stroke volume and cardiac output [9] and maximal sprinting performance [11]. Despite athletes’ preparation and competition periods usually having their own training emphases aiming to improve specific abilities [12], there is practically no scientific debate in the literature about how to periodize testing according to these emphases.

In general, improvements in untrained participants in all of these determinants have been observed with both low- and high intensity training: VO_2max_ [13], durability [8], fat oxidation capacity [14], lactate thresholds [4], cardiac output [15,16], economy of movement [17], and sprinting ability [18]. Although endurance training undoubtedly influences the modifications in these variables, a consensus regarding the impact of the frequency-duration-intensity triplet on these variables has not yet been reached.

The purpose of this study was to examine whether a lack of intensity could be compensated for by increasing volume, i.e., the duration and frequency of the exercises. Therefore, we compared the effects of a very high volume, very low intensity (LIT) cycling protocol with those of an intensive high intensity training (HIT). To gain a more comprehensive understanding of the impact of exercise intensity and volume, we examined the effects of HIT and LIT specifically on high intensity (sprint performance, second lactate threshold, aerobic capacity, maximal stroke volume) and low intensity (first lactate threshold, fat oxidation during exercise, acute recovery) performance categories.

## Materials and methods

### Participants

**Fig 1:**
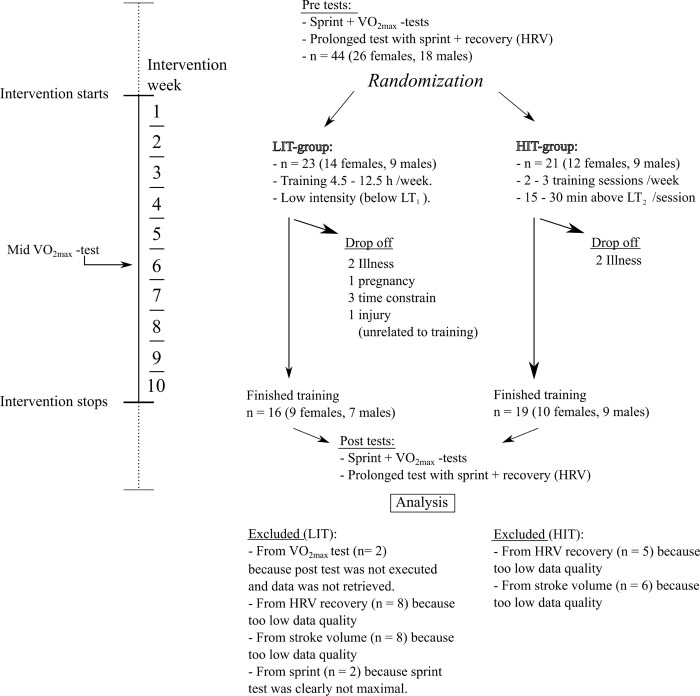
Flow chart of the study. *LIT* Low Intensity training group. *HIT* High Intensity Training group. *VO*_*2max*_ *test* Maximum oxygen uptake test. *HRV* Heart rate variability.

Recruitment started 1.6.2021 and ended 30.9.2021. Healthy sedentary or recreationally active untrained 18–40-year-old adults took part in the study, and their characteristics are in Table 1, with height and body mass (InBody 770, Biospace Ltd., Seoul, Korea) measured in the morning after at least 8 h fasting. Totally 35 (16 males, 19 females) of 44 implemented the exercise intervention (Fig 1). All participants provided a written informed consent. The study was approved by the Ethical Committee of the Central Finland Health Care District (8U/2020) compiled with the Declaration of Helsinki.

**Table 1:** Mean (SD) of the basic characteristics of the participants at the beginning of the study.

		Body mass index (kg/m^2^)	Age (y)	VO_2_max (ml/kg/min)
Low intensity training group	Female (n = 9)	25.9 (4.6)	32 (5)	35.1 (3.7)
	Male (n = 7)	27.7 (2.8)	34 (6)	40.9 (5.5)
	Combined (n = 16)	26.7 (3.9)	33 (6)	37.6 (5.3)
High intensity training group	Female (n = 10)	23.5 (3.4)	30 (5)	37.6 (5.0)
	Male (n = 9)	27.0 (2.6)	34 (5)	37.2 (6.4)
	Combined (n = 19)	25.1 (3.5)	32 (5)	37.4 (5.5)

*VO*_*2max*_ Maximal oxygen uptake.

### Training

A shortened description of training, tests and analysis is given, and a detailed one can be found elsewhere [8]. Participants were randomly divided into LIT (n = 16) or HIT (n = 19) cycling groups for 10 weeks. Weekly training load progressed individually, emphasizing volume progression. Progression was based on weekly ratio of perceived exertion (RPE); the easier the week was perceived, the greater the increase in volume. There were recovery weeks in the intervention weeks 3 and 7 to facilitate recovery. Using recovery weeks with lower training volume without alternating intensity maintains [19] or even enhance [20] endurance performance. From each exercise session, heart rate (Garmin Forerunner 945 and Garmin HRM-Pro belt, Garmin Ltd., Taiwan), power (Rally RK200, Garmin Ltd., Taiwan; or Wattbike Trainer, Wattbike Ltd., Nottingham, UK), and RPE was measured.

### Training intensity distribution

**Fig 2:**
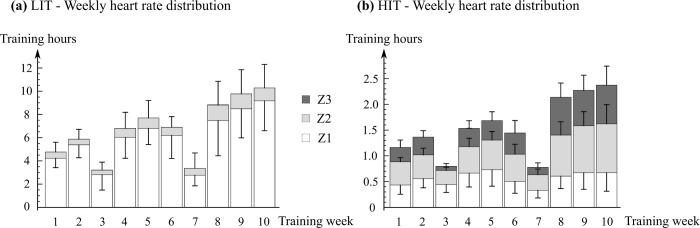
Mean (SD) weekly training heart rate distribution for (A) LIT, SD only for zones Z1 and Z2; (B) HIT. Notice the different y-axis in figures. *LIT* Low intensity training. *HIT* High intensity training. *Heart rate zones Z1* (below LT_1_); *Z2* (between LT_1_ to LT_2_); *Z3* (above LT_2_). *LT*_*1*_ First lactate threshold. *LT*_*2*_ Second lactate threshold.

Heart rates were distributed into the three zones: Z1 (below LT_1_), Z2 (between LT_1_ and LT_2_); Z3 (above LT_2_). Realized weekly training intensity distributions in the groups are shown in Fig 2. The total training HR intensity distribution in zones Z1–Z3 were 88 (18)% / 12 (17)% / 0 (0)% in the LIT group and 36 (10)% / 38 (2)% / 26 (11)% in the HIT group, respectively.

In the LIT group, the mean weekly training volume was 6.7 (0.8) h with a frequency of 4.8 (0.3) times. The mean RPE (0–10) was 2.7 (1.3) and the mean training intensity was 63 (4)% HR_max_. In addition, it was calculated that on average participants in the LIT group exceeded LT_1_ -power 21.0 (17.0) times weekly (30s average power). In the HIT group, the mean weekly training volume was 1.6 (0.2) h, frequency 2.4 (0.1) times, RPE 7.2 (1.9), and the training power in the work intervals was 117 (4)% of LT_2_ power.

Training in the LIT group had 3–6 weekly sessions mostly outdoors (3 did all sessions indoors, others 3%) each lasting 45–240 min and target power was below the power of the first lactate threshold (LT_1_). The mean realization of intensity during LIT was 63 (4)% HR_max_ or 87 (7)% HR_LT1_ or 47 (5)% VO_2max_ or 42 (5)% VO_2_Reserve. The HIT group had 2–3 weekly indoor sessions. In each session, they did 10 min warm up and 10 min cool-down at the power level of below 60 W. As the main part, they cycled long work intervals (3–7 min) with target power 110% of the power of the second lactate threshold (LT_2_) with at least 15 min worth of cumulative work time in a session.

### Measured variables

*Maximal graded test* was done three times: preceding the intervention, at the intervention week 6, and after the intervention. VO_2max_, LT_1_, LT_2_, and maximal aerobic power (P_max_) associated with VO_2max_ were measured. LT_1_ was defined as the lowest value of the lactate/VO_2_ -ratio, and the second lactate threshold (LT_2_) as a sudden and sustained increase in blood lactate concentration [21]. In addition, maximal stroke volume (SV_max_) was measured during the test by the noninvasive impedance cardiography (PhysioFlow PF-07 Enduro, Manatec Biomedical, France) with PF50 PhysioFlow electrodes. Other equipment included: ergometer (Monark LC4, Monark Exercise AB, Sweden), respiratory gas analyzer (Jaeger Vyntus TM CPX, CareFusion Germany 234 GmbH, Hoechberg, Germany), lactate analyzer (EKF-diagnostic GmbH Ebendorfer Chaussee 3, Germany), and HRM-pro or HRM-dual heart rate belt (Garmin Ltd., Taiwan).

*Sprinting power* was measured with 15 seconds Wingate -test (Monark 894E, Monark Exercise AB, Sweden), which is a reliable sprint test [22]. It was performed 35 minutes before the start of the graded test.

*Prolonged test*, lasting 3 h, was done preceding the intervention and again after the intervention. The participants were advised to have a standard meal 2.5–3 h before entering the laboratory. The absolute power was identical in the pre- and post-tests, and it was prescribed as 50% pre-VO_2max_. The actual measured intensity in the pre-test was 48 (4)% VO_2max_. Fat oxidation (g/min) was measured at 20–30 minutes into the test from gas exchange variables by the equation $1.67\times {\mathrm{VO}}_{2}(l/\min)-1.67\times {\mathrm{VCO}}_{2}(l/\min)$ [23]. During the first 20 minutes, the participants chose their preferred position and cadence (over 60 rpm), which were recorded and maintained during the subsequent 10-minute measurement slot in both pre- and post-tests. After the 10-minute measurement slot, participants could adjust position and cadence (> 60 rpm) freely. During the test, carbohydrates were given to cover 50% of theoretical energy expenditure. As nutrition affects the fat oxidation measurement, carbohydrate intake was started after the first 30 minutes. Immediately after the 3-h prolonged test, the participants did 15 seconds Wingate -test, after which the participants sat quietly for 15 minutes on a chair to record recovery heart rate variability (HRV), and analyzed with Kubios HRV Premium (Version 3.5.0, Kubios Oy, Kuopio, Finland).

### Heart rate variability (HRV)

In the HRV-analysis, low frequency (0.04–0.15 Hz) and high frequency (0.15–1.1 Hz) bands were combined into one total power band. The HRV samples were 3-minute subintervals during the recovery period at minutes 3, 6, 9, and 15. Only data points with effective data length of at least 90% were included in the final analysis. If participant had more than 2 missing data points of 8 possible ones, he/she was removed from final analysis. At total 8 in the LIT and 5 participants in the HIT group were removed. Of the rest, 12.5% of HRV data were missing. Multiple imputations were used to fill in missing data.

### Performance categories

We separated low and high intensity performance categories and included all “traditional” endurance training performance variables that were measured during the study.

*Low intensity performance category* was encompassed variables measured at or below LT_1_ -intensity: LT_1_ described both as absolute power as well as VO_2_-intensity relative to VO_2max_, fat oxidation at the prolonged test, and HRV recovery followed from the prolonged cycling and sprint -tests.

*High intensity performance category* included variables measured above the first lactate threshold: LT_2_ described both as absolute power as well as VO_2_-intensity relative to VO_2max_, VO_2max_, P_max_, sprinting power, and maximal stroke volume.

### Statistical analyses

Although the study question was included in the original study plan, the original sample size was calculated for the primary outcome of the previous study (rise in energy expenditure during prolonged cycling test) [8]. No sample size calculation was performed on the outcome measures of this study. Data is presented as mean (standard deviation). Statistical tests were calculated by 28.0 (SPSS Inc, Chicago, IL, USA) or by Mathematica 13.0 (Wolfram Research, USA). The Shapiro-Wilk test was used to examine the normality together with visual inspection. Change in fat oxidation failed to be normally distributed. The variables in the low- and high intensity performance categories were selected before performing the analyses. Before the final analysis, it was determined, applying Kurskal-Wallis, if the magnitude of changes in the variables differed between sexes. No differences were detected in any variables and therefore, females and males were analyzed in a combined group.

Between-group differences were tested with repeated measure ANOVA. If sphericity assumption failed, Greenhouse-Geisser correction was used. In the paired comparison 95% CI were calculated and t-test was used, except for fat oxidation where Wilcoxon test was utilized. The effect size (ES) of differences for the main variables were calculated with a corrected effect size Hedge’s g. Effect size for between-group differences was calculated by subtracting within-group effect sizes from each other [24]. Small, moderate, large, and very large effect size magnitudes for Hedge’s g were categorized as 0.20, 0.50, 0.80, and 1.2.

The variables were pooled in low- and high intensity performance categories by averaging z-score values, from which pooled p-value was calculated. Effect sizes were pooled following the suggestion by [24]: Cohen d_i_ ES→ Biseral r_i_ ES→ Fisher *Z_ri_*→ Taking average ${\bar{Z}}_{r}=Mean(Z_{ri})$ → Averaged biseral $\bar{r}$ ES → Averaged $\bar{d}$ ES → Averaged Hedge’s $\bar{g}$, where *i* is the different variables from which averages were taken.

## Results

**Fig 3:**
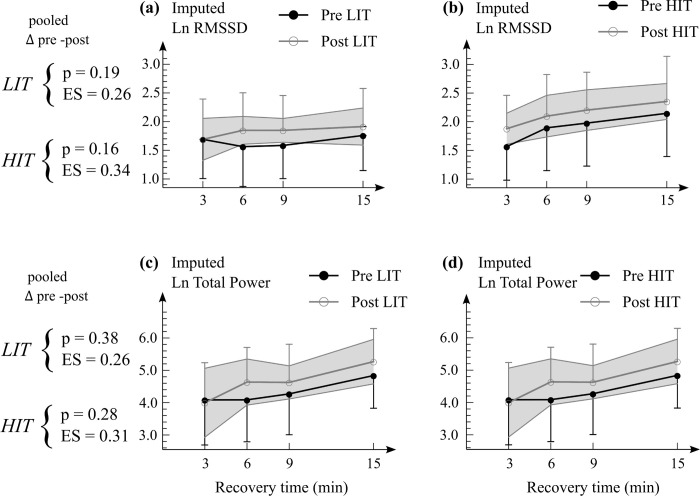
(A–B) Mean (SD) Ln of imputed RMSSD, (C–D) Ln of imputed total heart rate variability power during 15 min recovery after the prolonged test. Pooled p-values and ES were averaged from the measured time points to represent the average change. Sample sizes were n = 8 for the LIT and n = 14 for the HIT group. The gray area represents 95% confidence interval for the change in variable (i.e. if pre-value is outside gray area, the change in that time point is significant at p = 0.05 level). There were no time x group differences on average between the groups in LnRMSSD nor LnTotal power (p > 0.20). *LIT* Low intensity training group, *HIT* High intensity training group. *p* p-value. *ES* Effect size (Hedge’s g). *Ln* Natural logarithm. *RMSSD* Root mean square of standard deviation. *Total power* Combined low (0.04–0.15 Hz) and high (0.15–1.1 Hz) frequency bands.

*Low intensity performance* improved on average in the LIT group (pooled p = 0.01, pooled ES = 0.49), while no change was observed in the HIT group (pooled p = 0.20, pooled ES = 0.20) (Table 2). However, no time x group difference was detected on average (pooled p = 0.62, pooled ES = 0.28) (Table 2). The post-exercise recovery-HRV after the prolonged test with sprint was similar in the pre and post -conditions in both groups (Fig 3), and there were no time x group differences between the groups (pooled p = 0.87, pooled ES = 0.06).

**Table 2:** Low intensity performances in the LIT and HIT groups. Description as mean (SD) and change from pre to post as Mean (95% CI).

	Low intensity training group (n = 16)				High intensity training group (n = 19)				Between group time x group (pre to post)
	pre	mid	post	Change (pre to post)	pre	mid	post	Change (pre to post)	
Power (W) at LT_1_	98 (28) (n = 15)	109 (26) (n = 15)	115 (26) (n = 15)	16 (8–25)	96 (29)	105 (32)	111 (32)	15 (5–25)	p = 0.93 ES = 0.11
				p < 0.001 ES = 0.60				p = 0.005 ES = 0.49	
Oxygen consumption (%VO_2max_) at LT_1_	54.8 (5.8) (n = 14)	55.7 (5.5) (n = 14)	58.1 (5.5) (n = 14)	3.4 (0.2–6.5)	55.9 (4.2)	53.8 (5.0)	55.8 (4.9)	-0.1 (-2.8–2.6)	p = 0.09 ES = 0.60
				p = 0.04 ES = 0.57				p = 0.93 ES = -0.02	
Fat oxidation (mg/min) at low intensity exercise	155 (58)		187 (84)	31 (-5–67)	168 (109)		174 (79)	6 (-40–52)	p = 0.86 ES = 0.36
				p = 0.06 ES = 0.42				p = 0.36 ES = 0.06	

*LT*_*1*_ First lactate threshold. *ES* Effect size. *p* p-value.

*High intensity performance did* not improve on average in the LIT group (pooled p = 0.34, pooled ES = 0.05), while it enhanced on average in the HIT group (pooled p = 0.007, pooled ES = 0.48) (Table 3). There was a time x group difference between the groups on average (pooled p = 0.02, pooled ES = 0.46) (Table 3).

**Table 3:** High intensity performances in the LIT and HIT groups. Description as mean (SD) and change from the pre to posttest as mean (95% CI).

	Low intensity training group (n = 14)				High intensity training group (n = 19)				Between group time x group (pre to post)
	Pre	Mid	post	Change (pre to post)	pre	mid	post	Change (pre to post)	
Power (W) at LT_2_	162 (42) (n = 15)	164 (39) (n = 15)	168 (37) (n = 15)	6 (-1–13) p = 0.14 ES = 0.14	158 (42)	170 (40)	178 (38)	21 (15–27) p < 0.001 ES = 0.51	p = 0.007 ES = 0.37
Oxygen uptake (% VO_2max_) at LT_2_	77.6 (6.5)	76.0 (4.5)	77.8 (5.2)	0.1 (-3.4–3.6) p = 0.95 ES = 0.03	79.1 (5.8)	77.4 (3.4)	78.9 (4.4)	-0.2 (-2.9–2.6) p = 0.90 ES = -0.03	p = 0.97 ES = -0.06
VO_2max_ (l/min)	2.90 (0.62)	2.96 (0.61)	2.93 (0.51)	0.02 (-0.11–0.16) p = 0.71 ES = 0.04	2.76 (0.55)	2.97 (0.61)	3.08 (0.59)	0.32 (0.22–0.42) p < 0.001 ES = 1.55	p < 0.001 ES = 1.51
P_max_ (W)	217 (47)	223 (49)	228 (45)	11 (3–19) p = 0.008 ES = 0.23	212 (48)	231 (49)	239 (47)	27 (21–33) p < 0.001 ES = 0.55	p < 0.001 ES = 0.32
Max stroke volume (ml)	126 (21) (n = 8)		120 (27) (n = 8)	-6 (-25–13) p = 0.50 ES = -0.22	127 (41) (n = 13)		135 (42) (n = 13)	8 (-3–20) p = 0.13 ES = 0.20	p = 0.14 ES = 0.42
15 s sprint (W)	650 (152)		647 (147)	-3 (-13–7) p = 0.57 ES = -0.02	635 (161)		656 (166)	21 (10–32) p < 0.001 ES = 0.13	p = 0.003 ES = 0.14

*LT*_*2*_ Second lactate threshold. *VO*_*2max*_ Maximal oxygen uptake. *P*_*max*_ Power associated to VO_2max_. *ES* Effect size. *p* p-value.

Body mass index increased in the HIT group (+0.5 kg/m^2^, 95% CI: 0.2–0.7 kg/m^2^, p<0.001, ES = 0.88), but not in the LIT group (+0.1 kg/m^2^, 95% CI: -0.2–0.5 kg/m^2^, p = 0.35, ES = 0.23) with no time x group difference (p = 0.09, ES = 0.65).

## Discussion

The aim of the present study was to investigate whether a lack of intensity could be compensated for by increasing volume, i.e., the duration and frequency of the exercises in low and high intensity performances. The main finding was the specificity of training. When the total training hours were moderately high (averaging 10.3 ± 1.8 h in the last week), very low intensity cycling training (63% HR_max_, or 87% HR_LT1_, or 42% VO_2_Reserve, or 47% VO_2max_) enhanced pooled low, but not high, intensity performance of untrained participants. Conversely, HIT improved pooled high intensity performance without concurrent improvements in low intensity one. This highlights two points. First, training volume alone cannot fully compensate for very low intensity of exercise even for untrained people, as averaged high-intensity performance adaptations did not occur. Second, the intensity threshold for low intensity performance is lower than for high intensity performance, and therefore, the success of low intensity exercise intervention cannot be monitored by high intensity performance tests alone.

### Low intensity performance

Surprisingly, HIT did not lead to distinct improvements in low intensity performance, although there was no difference between the HIT and LIT groups. This finding is unexpected, since HIT has been previously reported to be as effective as LIT in enhancing low intensity performance, including the first lactate threshold [16,25] and fat oxidation [14].

### First lactate threshold and fat oxidation

Both LIT and HIT improved cycling power at the first lactate threshold, which is usual observations in literature [16,25]. However, only LIT improved LT_1_ relative to VO_2max_. Usually lactate thresholds are linked to peripheral factors, such as general oxidative capacity of a muscle, fiber type, capillary density, and mitochondrial density [26–28]. These peripheral adaptations often coincide with a shift from glycogen to lipid utilization [26,27]. However, in the present study, this joint development was not found, as fat oxidation was not unambiguously altered. This discrepancy might be attributed to at least two factors. First, our sample size might have been insufficient to detect the increased lipid utilization. Secondly, the participants in our study were not in a fasted state, and the previous meal may influence fat oxidation values at exercise [29].

### HRV recovery

Neither LIT nor HIT had an impact on HRV recovery in the present study. This is partially supported by the previous literature. Endurance training can accelerate post-exercise HRV recovery following endurance exercises by improving parasympathetic reactivation [10,30]. However, these improvements have typically been observed after submaximal exercises. On the other hand, post-exercise HRV recovery following maximal efforts, such as sprint in our study, is usually unaffected by exercise interventions [31].

This could be attributed to the fact that post-exercise HRV can serve as an indicator of exercise intensity, with the anaerobic contribution playing a significant role in determining the extent of parasympathetic reactivation. Thus, irrespective of training status, a maximal effort characterized by equal anaerobic contribution could result in a comparable post-exercise HRV state.

### High intensity performance

#### VO_2max_ and P_max_

Although HIT can induce slightly greater enhancement in VO_2max_ than LIT [13], also LIT elicits clear positive adaptations in P_max_. Improved VO_2max_ is mostly explained by central adaptation, particularly improved cardiac output [32]. This, in turn, is mostly explained by increased blood volume, as structural and functional changes within myocardium takes longer than a few weeks [32]. Therefore, it seems plausible to suggest that the very low intensity training employed in our study was inadequate to elicit a substantial increase in blood volume, although increase in blood volume does not necessary lead to improved maximal stroke volume and hence VO_2max_ [16].

Although VO_2max_ did not improve following LIT, the aerobic power P_max_ improved slightly. This is in practice often more important than improved oxygen uptake. After all, although heavily linked, aerobic performance ultimately depends on external power produced rather than internal energy expenditure. Peripheral adaptations might be enough to improve cycling capacity without VO_2max_ improvement [28]. Although economy of cycling movement is difficult to improve, it may be possible for untrained participants [33]. Enhanced anaerobic capacity could also result in improved P_max_. Although it is possible to improve anaerobic performance through LIT [18], it did not materialize in the present study, as evaluated by unchanged 15-second sprinting ability. It is also possible, that unaccustomed participants learned during the intervention how to implement graded test.

It has been proposed that the relatively modest stimulus elicited by low intensity endurance exercise may be counterbalanced by extending the duration of the exercise [34]. However, this may not capture the full complexity of the matter. A meta-analysis suggests a possible existence of a critical threshold of intensity, below which training fails to improve aerobic capacity [2]. Specifically, for untrained individuals with an initial aerobic capacity exceeding 40 ml/kg/min, this critical intensity required to improve aerobic capacity would be around 50% of VO_2max_ [2]. In the present study, the baseline aerobic capacity of the LIT group was close to this threshold (40.9 ml/kg/min for males, 34.5 ml/kg/min for females), while their training intensity averaged 47% VO_2max_. Consequently, our findings provide empirical support for the idea that intensity plays a pivotal role in driving enhancements in VO_2max_ and variables in high intensity performance category, in general.

### Second lactate threshold and sprint

The second lactate threshold was increased exclusively in the HIT group, despite the fact that both thresholds are associated to the peripheral factors. A similar phenomenon, in which HIT exhibits a greater emphasis on the development of LT_2_ compared to LIT, has been reported previously [25]. The observed different response between the present HIT and LIT groups may be attributed to their distinct mechanisms of muscle fiber activation. LT_2_ represents the intensity level at which almost all fast twitch fibers are recruited [35]. While prolonged LIT sessions can recruit also fast twitch fibers [35], the LIT group likely focused mainly on training slow twitch fibers, whereas the HIT group recruited a broader range of muscle fibers in each session.

HIT, but not LIT, improved sprint. That could be explained by enhanced muscle strength, which has been observed to occur after high intensity endurance training in untrained participants [36].

### Time courses of adaptations

The intervention of the present study included 8 weeks training and two recovery weeks. This timeframe has usually been sufficient for noticeable endurance adaptations. Peripheral adaptations in oxidative energy production and metabolism occur rapidly. Indeed, within less than two weeks, mitochondrial density can increase up to 30% [37] and fat oxidation 10% [38]. On the other hand, central adaptations are typically of lesser magnitude and slower, apart from plasma expansion, which may be up to +20% in three days [39]. While cardiac remodeling may not be visible after six weeks [32], it becomes apparent after three months, characterized by ~10–15% greater left ventricular mass and mean wall thickness [40]. In conclusion, if the training introduced in the present study were to induce endurance adaptations, they would likely have become apparent during the intervention period.

### Training

The implementation of the training was successful. The LIT group did not exceed LT_1_ power, and the work intervals in the HIT group were on average +17% above LT_2_ power. Therefore, heart rate drift and kinetics presumably explain why HR distribution was not more LIT emphasized in the LIT group, or more polarized in the HIT group. For untrained participants, the volume of training in the LIT group was large, although not exceptionally large. The total volume averaged 6.7 h /week and the last three weeks 9.6 h/week. For example, in a meta-analysis [13] none of the 21 included studies that contained LIT/MIT exceeded 5 weekly hours. Moreover, studies comparing different volumes of LIT usually contain 1–5 weekly hours [41–44]. In line with the present study, these comparable studies also suggest that adding LIT volume above certain threshold does not offer advantages in VO_2max_ improvements. Further, excess amount of LIT, such as 6 h daily skiing [45,46], has not impacted VO_2max_, while peripheral adaptations, although not excessive, occurred.

### Applications to practical training

Athletes engage in periods of LIT- or HIT-focused training [12], although not necessarily as fully dedicated as in this study. Surprisingly, periodizing testing is not a heavily debated subject. Our results would suggest distinct test patterns for these types of LIT and HIT emphasized periods. It would not be even expected that high intensity variables would improve during the emphasized LIT period, and conversely with the HIT period.

Our results would also indicate that an athlete wanting to excel in very long steady state competitions have straight benefits from high volume LIT. These include e.g. ironman triathlon and ultradistance running, where competition intensity is near LT_1_, and fat oxidation and relative LT_1_ intensity have direct effect on competition. On the other hand, for short-distance endurance athletes with competition intensity above LT_2_, such as middle distance runners, cycling time triallists or 400 m swimmers, our results would indicate a direct benefit from emphasized HIT.

However, real life situation is not so straightforward. First, high VO_2max_ would be warranted also in very long-distance athletes, as it enables also high LT_1_, so they should also be engaged in HIT in some part of training year. On the other hand, although directly LIT does not seem to offer help for short-distance athletes, it may offer indirect benefits. For example, improved fat oxidation leads to spared glycogen stores during exercises and thus faster glycogen replenishment after exercise sessions.

### Limitations

The sample size was not designed to meet the requirements of this study. Additionally, dietary monitoring was not incorporated, although diet could have impact on potential endurance adaptations, especially fat oxidation [47]. Further, insufficient caloric intake may lead to insufficient recovery and subsequently compromise endurance training adaptations [48]. However, participants were encouraged to eat sufficiently to meet the potentially increased energy demands caused by the training. This was successful, although not in terms of weight management, in that weight increased rather than decreased during the intervention. Additionally, conducting the prolonged test without fasting may have contributed to the fat oxidation results. However, the previous meal was standardized, and fat oxidation represents only one aspect in low intensity performance, and its influence alone cannot alter the observed results.

The baseline measurements may influence the adaptations due to regression to the mean -phenomenon. The most efficient ways to minimize the regression to the mean is to have a control group, to make duplicate measurements of the initial values, and to use surrogate measurements [49]. However, the most potential problem with regression to the mean is when the treatment is assessed in subgroups based on pre-treatment values (i.e. ‘low’ and ‘high’ level -groups) and when the intra-individual SD is large compared to inter-individual SD [49]. Although regression to the mean is always present, our study may not have a major problem with it, as the groups were not classified by pre-treatment values, and e.g. intra-individual SD of VO_2max_ (typically around 4% [49]) was not particularly high compared to inter-individual SD of ~20%. Finally, the present study used many surrogate measurements, which formed low- and high intensity performance variables.

The training dose between the groups were equalize with a rare metric based on a weekly RPE. However, this idea merely extends the well-accepted session RPE [50] to a weekly level. This allows scientifically controlled individual progression to the training. The incorporation of additional low and high intensity performance variables would have contributed to a more comprehensive understanding of the findings. We did not assess the economy of movement, as endurance training has typically only minimal effect on cycling economy [51]. Further, the present study protocol did not allow for the implementation of the fatmax concept since the graded test was not conducted at fasted state and step lengths were too short. Another aspect of interest would be durability, which refers to the ability to resist fatigue in a prolonged submaximal effort [8]. However, we have already reported, based on the same intervention, that both LIT and HIT improved durability [8]. Adding durability result from [8] to the pooled low intensity performance would slightly lower the p-value of the HIT group (from p = 0.20 to 0.13), but it would not change the conclusion of the present study. Two interesting high intensity performance variables could have been time trial performance and maximal enzyme activities, which would have provided more in-depth explanations for the observed results.

## Conclusions

The present study showed training specificity on pooled low and high intensity performances. HIT improved high intensity performance but had no impact on low intensity performance. On the other hand, the effect of very high volume of very low intensity training (63% HR_max_) was the opposite. These findings have two implications. Firstly, very low intensity cannot be fully compensated by high training volume even for untrained participants, as pooled high intensity performance determinants were unaltered. Secondly, minimal threshold for low intensity training zone depends on the specific performance aspect targeted for improvement. Based on the present findings, the minimal intensity required to enhance high intensity performance (including sprint, VO_2max_, P_max_, maximal stroke volume, and the second lactate threshold) is higher than the intensity required to improve low intensity performance (including fat oxidation, HRV recovery, and the first lactate threshold). Consequently, evaluating the effectiveness of LIT on endurance performance cannot be solely determined by high intensity performance tests.

### Practical applications

There are two practical applications of this study. First, in order to see the benefits of LIT on endurance performance, one should consider low intensity performance tests, as high-intensity performance tests (e.g., time trial or VO_2max_ test) may not provide a comprehensive picture of the benefits of LIT. Second, very high volume alone cannot compensate for the lack of sufficient intensity for the overall development of endurance performance.
